# Editorial: Right ventricular failure: current strategies and future development

**DOI:** 10.3389/fcvm.2024.1388501

**Published:** 2024-07-05

**Authors:** Marco Follis, Roberto Lorusso

**Affiliations:** ^1^ Department of Cardio-Thoracic and Vascular Surgery, Klinikum Braunschweig, Braunschweig, Germany; ^2^ Department of Cardio-Thoracic Surgery, Maastricht University Medical Centre, Maastricht, Netherlands; ^3^ Cardiovascular Research Institute Maastricht (CARIM), Maastricht, Netherlands

**Keywords:** right ventricular failure, ECLS, mechanical circulatory support, VAD, heart failure

**Editorial on the Research Topic** Right ventricular failure: current strategies and future development

Acute right ventricular failure (RVF) is a clinical condition characterized by a quick functional compromise of the right ventricle (RV) and its inability to effectively pump blood through the right ventricle outflow tract into the pulmonary circulation in a setting of adequate preload that leads to a cascade of hemodynamic and clinical consequences. It can be the result of a plethora of different causes, including pathological processes that increase native pulmonary pressures, pathology to the native right heart cardiac valves, right ventricular ischemia, constrictive pericarditis or cardiac tamponade, or as a complication of left-sided heart failure (1). The significance of RVF lies in its increased morbidity and mortality and has been shown to be associated with poor clinical outcomes and increased healthcare utilization (2). In the past, the right ventricle and the related pathologies have been somewhat neglected “the forgotten ventricle”, but its relevance and challenges in patient management and outcome have received increasing attention (3). With the development of new medical strategies and new support devices for the right ventricle, there have been significant advancements in treating acute RVF. Despite these advancements, challenges in managing acute RVF persist, particularly in diagnosing RVF and identifying the optimal timing and selection of therapies. The articles in this research topic address some of these critical issues and provide readers with new insights into the current state of management RVF, as well as emerging therapeutic strategies.

Ro et al. delve into the complexities of diagnosing RVF, highlighting the critical need for timely identification to ensure effective management and improved clinical outcomes. Echocardiography remains the primary diagnostic tool because of its accessibility, non-invasive nature, and cost-effectiveness. It plays a vital role in detecting the root causes of right ventricular pathology and monitoring treatment responses (4, 5). Advanced echocardiography techniques, such as 3D, speckle-tracking, and stress echocardiography, enhance diagnostic precision by offering detailed insights into RV function.

Cardiac magnetic resonance (CMR) is deemed the gold standard for detailed RV imaging due to its ability to provide precise volumetric and functional assessments without radiation exposure (6, 7). However, its high cost and limited availability hinder routine use. In the future, AI-enhanced CMR techniques promise to improve the reproducibility and accuracy of RV assessments.

Radionuclide imaging, initially used for left ventricular function studies, has been adapted for RV assessment, especially in pre-LVAD evaluations, because RVF is a common complication of LVAD implantation (8). This imaging technique offers accuracy comparable to CMR (9).

Despite declining use, pulmonary artery catheter-based hemodynamic assessment has resurged recently, proving useful for differential diagnosis and continuous cardio-circulatory monitoring (10).

The review also clarifies the importance of emerging biomarkers, such as natriuretic peptides, troponins, ST2, and Galectin-3, in diagnosing RVF.

In addition to diagnostic challenges, management of acute RVF also presents its own set of difficulties. Monteagudo-Vela et al. reviewed the current multifaceted strategies essential for effective RVF treatment, highlighting three core approaches: Volume optimization, restoration of perfusion pressure and improvement of myocardial contractility. Treatment aims to fine-tune the hemodynamic balance, employing diuretics to mitigate volume overload, norepinephrine and epinephrine to enhance end-organ perfusion pressure, and inotropes such as dobutamine, milrinone, and levosimendan to augment myocardial contractility (3, 11, 12). For patients unresponsive to medical therapies, short-term or long-term mechanical circulatory support (MCS), whether percutaneously or surgically implanted, may become necessary.

Recognizing the right ventricle's remarkable capacity for recovery, percutaneous short-term support is emerging as a highly promising therapeutic option for RV dysfunction. James et al. describe the evolution of percutaneous right ventricular assist devices (pRVAD), drawing on various institutional experiences. While existing data remains limited, primarily from retrospective single-center studies with small patient cohorts, the advancements in pRVADs show promising outcomes (13–15). These outcomes are often comparable, if not superior, to those of surgically implanted RVADs, offering fewer complications and shorter hospital stays.

As the use of MCS continues to increase worldwide, Kuroda et al. provide a comprehensive review of all current MCS devices that are available for RVF, focusing on their application and efficacy as well as possible combinations in the case of biventricular failure (ECPELLA and BiPella). They can be categorized by function: direct RV bypass, indirect RV bypass, and systems for chronic RV support. “direct RV Bypass” devices that are currently available include Impella RP (micro axial flow that delivers blood from the right atrium (RA) directly into the pulmonary artery (PA)) and LifeSPARC pump with Protekduo cannula (extracorporeal continuous flow pump with a single dual-lumen cannula that also directly bypasses the RV by delivering blood from the RA directly to the PA with the bonus of being upgradable to an OxyRAD through the simple addition of an Oxygenator). “Indirect RV Bypass” is constituted by V-A ECMO (extracorporeal centrifugal flow pump with two cannulas, venous and arterial, that aspirates venous blood from the RA, passing it through an oxygenator and remitting the oxygenated blood into the arterial system in a retrograde fashion).

For chronic support, off-label use of HeartMate 3 and Total Artificial Hearts like SynCardia and Aeson is possible with the caveat that these devices were designed for a high-pressure system and not tailored for RV physiology and hemodynamics. These devices require surgical access at the time of both implantation and explantation, and unfortunately, long-term data regarding their use in RVF is still lacking and incomplete. Emphasizing the necessity of early, precise interventions and the selection of appropriate devices, it is important to underline the potential benefits of dedicated, durable RVADs and improved percutaneous device mobility for patient rehabilitation and survival.

Brown et al. observed in their review that RV dysfunction is a significant concern in ARDS patients receiving V-V ECMO, often resulting from elevated pulmonary vascular resistance (PVR). V-V ECMO supports gas exchange and allows for lung-protective ventilation, but persistent RV dysfunction necessitates careful management and monitoring. Therapeutic measures include vasopressors, inotropes, prone positioning, and lung-protective ventilation to improve oxygenation and reduce RV afterload. Severe cases may require transitioning to V-A ECMO, intra-aortic balloon pumps, or RV assist devices to provide direct cardiac support and improve patient outcomes.

The evolution in minimally invasive percutaneous techniques has expanded our ability to treat conditions like tricuspid regurgitation more safely and with favorable outcomes. Albertini et al. studied the effects of transcatheter tricuspid leaflet repair on RV remodeling in patients with functional tricuspid regurgitation (fTR). Traditional surgical interventions are often high-risk and unsatisfactory, prompting interest in less invasive techniques like the TriClip™ and PASCAL systems. These transcatheter approaches allow for the repair of the tricuspid valve without the need for open-heart surgery, reducing procedural risks and facilitating faster recovery, showing early success in reducing RV volumes, RV remodeling, and improving function, mirroring surgical outcomes (16, 17).

While our understanding of RVF pathophysiology and hemodynamics has come a long way in the last decade we are still very limited in our treatment of this disease. Our aim should be to focus on the advancement and innovation of new medical and mechanical support strategies, with a particular emphasis on long-term care. These strategies should be tailored specifically to treat the dysfunctional right ventricle, taking into consideration its unique anatomy and physiology.
